# Profile: Derek Summerfield – politics and psychiatry

**DOI:** 10.1192/pb.bp.117.056556

**Published:** 2017-10

**Authors:** Julia Bland

**Figure:**
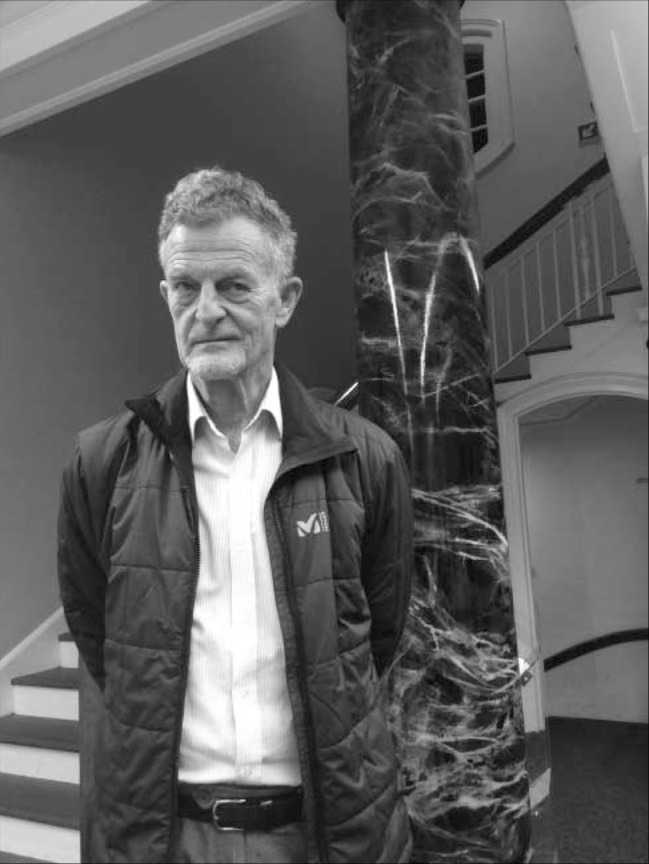


Preparing for this interview, I was aware of Dr Summerfield's political commitment. After all, he was the principal psychiatrist for Freedom from Torture (Medical Foundation for the Care of Victims of Torture) from 1991 until 2000. He is an outspoken critic of what he describes as the complicity of the Israeli Medical Association (IMA) and Israeli doctors posted to interrogation units in relation to the torture of Palestinian refugees, which has led to him receiving many accusations of anti-Semitism and becoming the subject of a libel suit by the President of the IMA. He is a member of the Critical Psychiatry Network. Before training in psychiatry, he worked as a government medical officer in Zimbabwe to ‘repay his debt’ to Africa. This was clearly going to be an encounter with a man of the left, passionate about social justice.

## The personal and the political

I was curious about how Dr Summerfield sees the personal and the political. Can they ever be separated? What exactly is the role of the psychiatrist in relation to social justice? Should what I will call ‘ethico-political awareness’ be part of psychiatric training, given the huge relevance to mental health of the context in which our patients live?

If senior psychiatrists should be leading the profession, can this ethico-political thinking be encouraged by example only, or more explicitly within the syllabus? And how does Derek Summerfield see his role within the profession? Is he an individual firebrand, grit in the oyster, provocateur, thinking iconoclast, leading by example and seeking influence via publication, or does he have a more intergenerational mission? (It emerges that he has recently canvassed the juniors at the Maudsley Hospital and recommended they should not comply with trust training in relation to the controversial government Prevent strategy of 2015, intended to detect early radicalisation in pupils, students or patients, which he regards as outrageous state intrusion into clinical confidentiality and a form of McCarthyism.)

More widely, in the recent climate of junior doctor strikes, dire warnings from the British Medical Association, hot controversy over the National Health Service as a public service, what, if any, are the political obligations of (a) doctors in general and (b) psychiatrists in particular? I got my answer loud and clear in a note he sent me a few days after the interview:

‘There is the doctor as doctor, but also (ethically imperative) the doctor as sentient citizen … To do with a fundamental duty to attend not just to the particulars of the patient sitting in front of us, but also to the political and socioeconomic factors that produce distress and disease. Doctors have largely not done this. End of sermon …’ .

Dr Summerfield is actually not preachy: he is amusing, not pompous, but self-deprecating – yet utterly (and perhaps unfashionably) serious. Of course he knows that not all doctors or psychiatrists share his views. Indeed, he is realistic:

‘With exceptions, doctors are not politically engaged. The average doctor doesn't have a social activist bone in his body… as a profession we are closer to those in power than those without it… medicine is basically an entrenched profession, and we behave like one … after the revolutions in places like Cuba, Mozambique, the vast majority of doctors left the country …’ .

I wonder how he was viewed by the international and non-homogeneous group of South London and Maudsley NHS Foundation Trust (SLAM) junior psychiatrists when they received his open letter about the Prevent strategy training. Is he admired as a model of the thinking critical psychiatrist, or dismissed as eccentric or even dangerously insurrectionist?

Derek Summerfield was born 68 years ago in Cape Town, South Africa, into a non-medical family, but grew up in Bulawayo, the second city, after Harare, of Zimbabwe (then Rhodesia). He studied physics initially (‘the headmaster persuaded bright White boys to do science’), then worked in a shipping company in Cape Town, and did some teaching. He always wanted to be a journalist (and now thinks he'd do law and anthropology if he was starting out again), but nevertheless began medicine, aged 24, at St Mary's Medical School in Paddington, London.

‘I failed everything, and nearly left in the first year. I had no interest or feel for science in the preclinical years, but when I got onto the wards I felt an intellectual thrill at last.’

He did house jobs (i.e. FY1) in Oxford and Cambridge, and went on to become a medical registrar with MRCP Part 1. During this period he did several stints in Zimbabwe during and after the civil war, no walk in the park. In Chiredzi, in the war-ravaged rural south-east, there were 200 beds and 2 doctors. He was the paediatrician, Vith 50 desperately sick kids at any one time … 5 of whom died each day’. He saw up close and personal the effect of poverty on health: ‘even measles was 80–100 times more fatal in these malnourished kids’.

He finally decided on psychiatry in 1982, now aged 34, and went to St George's Hospital. While working there he met a beautiful and much younger woman, now a consultant clinical psychologist and psychoanalyst at the Tavistock, Francesca Huhne. They have a 20-year-old daughter, who is reading History at university. He and Francesca subsequently studied war-wounded men from both sides of the civil war in Nicaragua together. He has been a consultant to Oxfam, and for many years was a research associate at the Refugee Studies Centre, University of Oxford.

He joined Freedom from Torture (Medical Foundation for the Care of Victims of Torture) full-time in 1991, where he treated around 800 patients, often seeking asylum in the UK: Bosnians, Sri Lankans, Iraqis, Turkish Kurds, Africans and Palestinians, with 95% of consultations requiring interpreters. But with his lifetime history of independent thinking and acting on principle, this appointment did not end amicably: he was ‘asked to leave’ Freedom from Torture in 2000 because the organisation was concerned that his widely published questioning of the category of posttraumatic stress disorder (PTSD) might deter funders.^1^ He sees the diagnosis of PTSD in the context of refugees as the ‘pseudo-scientific pathologising of people affected by war … the medicalisation of their situation diminishes the importance of work and the rebuilding of social networks … the broken social world is the lot of the asylum seeker’.^2^

After leaving Freedom from Torture, he contacted Dr Maurice Lipsedge, who suggested that he should consider applying for his job in SLAM, from which he was retiring. This was in the HIV mental health team, initially part time, then full time.

The HIV team at its zenith had four psychologists and acted as a community mental health team with an HIV focus. He enjoyed the interest of the ‘two different groups of patients … African women and British gay men’. The service was greatly reduced in 2016, owing to both financial constraints and the mainstreaming of HIV. He accepts this process as reasonable, given the changes in HIV treatment and prognosis, with life expectancy being almost normal now, if the medicines are taken reliably. (And many African women don't always do this, and he would like to understand why.)

He has no immediate plans to retire and is busy with a variety of work outside the ‘day job’. He's involved with the new medical school in Bulawayo, his home town, helping with the undergraduate curriculum, and will teach there: ‘I still feel a debt to Africa’. He advises Oxfam: In the 80s they suddenly got very interested in the mental health of victims of war‘. Derek Summerfield helped evaluate their projects in Bosnia, and steered Oxfam away from a PTSD-centred approach to victims of that war: ‘I persuaded them not to go down the counselling route … if you ask Muslim adolescents in Tuzla what they want, they want a bit of normality. They actually requested some fabric so they could put on a fashion show’.

## Problem with the concept of global mental health

He has published widely from the standpoint of a fundamental opposition to the medicalisation of human distress and the assumption that Western psychiatry is universally valid, which he sees as a kind of cultural imperialism.^3,4^

He offers a critique and deconstruction of international attitudes to the mental health of victims of war.^2^ He stresses how the global effects of wars are largely experienced by people living in poverty, and he sees the danger of the PTSD diagnosis as pathologising of the individual rather than attending to the effects of poverty and the need for reconstruction of the social fabric.^2^

He makes a more general critique of the concept of ‘global mental health’, highly critical of ‘taking depression into a country which has no such concept, followed by the marketing of antidepressants … Western cultural values parading as medical facts’.^3,4^ His critique extends to the cultural relativism of psychiatric diagnoses in general: as if disease had an objective existence, independent of the gaze of the diagnostician.

The origin of PTSD as a diagnosis was for disturbed Vietnam war veterans in the USA with the benign intention of lobbying to get decent care for them by emphasising the traumatogenic nature of war, thus legitimising a position of victimhood, moral exculpation and receipt of disability pensions. In Summerfield's view, the development of this concept of PTSD needs to be understood in the wider social context of the ‘rise of expressive psychologically minded individualism, personal rights, entitlement and grievance,’ as opposed to the previously socially sanctioned stiff upper lip self-management of trauma. Now PTSD has become a ‘certificate of impairment’. It is the only diagnosis which contains its aetiology within itself, while in fact, he claims, premorbid factors such as psychiatric history or a negative thinking style are more important than the actual event itself in the aetiology of symptoms.^1^

Conflating normality and pathology ‘devalues the currency of true illness’.

He is an old fighter, brave and almost proud of his battle scars: ‘No-one has been called an anti-Semite in the medical press more than me’. He feels the campaign he convened against medical complicity with torture in Israel is the ‘best thing I've done in my career’. So the life and views of Derek Summerfield are a challenge to all doctors, even those with different political views: is it acceptable for doctors to absent themselves from political engagement as being outside their remit, or are we ethically obliged to be properly aware of the sociopolitical context of our work? People with unflinching integrity have always made others uncomfortable: consider the unpopularity of Jesus overturning stalls in the temple market.

If we take our ethical obligations as doctors seriously, we need intellectually rigorous and dogged colleagues like Derek Summerfield to point up our innate conservatism and political passivity.
